# Phase 3 Multicenter Study of Revusiran in Patients with Hereditary Transthyretin-Mediated (hATTR) Amyloidosis with Cardiomyopathy (ENDEAVOUR)

**DOI:** 10.1007/s10557-019-06919-4

**Published:** 2020-02-15

**Authors:** Daniel P. Judge, Arnt V. Kristen, Martha Grogan, Mathew S. Maurer, Rodney H. Falk, Mazen Hanna, Julian Gillmore, Pushkal Garg, Akshay K. Vaishnaw, Jamie Harrop, Christine Powell, Verena Karsten, Xiaoping Zhang, Marianne T. Sweetser, John Vest, Philip N. Hawkins

**Affiliations:** 1 Johns Hopkins Hospital, Baltimore, MD USA; 2 Present Address: Medical University of South Carolina, Charleston, SC USA; 3 Department of Cardiology, University of Heidelberg, Heidelberg, Germany; 4 Mayo Clinic, Rochester, MN USA; 5 Columbia University Medical Center, New York, NY USA; 6 Brigham and Women’s Hospital, Boston, MA USA; 7 Cleveland Clinic, Cleveland, OH USA; 8 National Amyloidosis Centre, Division of Medicine, UCL Medical School Royal Free Hospital Rowland Hill Street, NW3 2PF, London, UK; 9 Alnylam Pharmaceuticals, Cambridge, MA USA

**Keywords:** ATTR amyloidosis, Cardiomyopathy, RNA interference, Revusiran

## Abstract

**Purpose:**

The Phase 3 ENDEAVOUR study evaluated revusiran, an investigational RNA interference therapeutic targeting hepatic transthyretin (TTR) production, for treating cardiomyopathy caused by hereditary transthyretin-mediated (hATTR) amyloidosis.

**Methods:**

Patients with hATTR amyloidosis with cardiomyopathy were randomized 2:1 to receive subcutaneous daily revusiran 500 mg (*n* = 140) or placebo (*n* = 66) for 5 days over a week followed by weekly doses. Co-primary endpoints were 6-min walk test distance and serum TTR reduction.

**Results:**

Revusiran treatment was stopped after a median of 6.71 months; the study Sponsor prematurely discontinued dosing due to an observed mortality imbalance between treatment arms. Eighteen (12.9%) patients on revusiran and 2 (3.0%) on placebo died during the on-treatment period. Most deaths in both treatment arms were adjudicated as cardiovascular due to heart failure (HF), consistent with the natural history of the disease. A post hoc safety investigation of patients treated with revusiran found that, at baseline, a greater proportion of those who died were ≥ 75 years and showed clinical evidence of more advanced HF compared with those who were alive throughout treatment. Revusiran pharmacokinetic exposures and TTR lowering did not show meaningful differences between patients who died and who were alive. Revusiran did not deleteriously affect echocardiographic parameters, cardiac biomarkers, or frequency of cardiovascular and HF hospitalization events.

**Conclusions:**

Causes for the observed mortality imbalance associated with revusiran were thoroughly investigated and no clear causative mechanism could be identified. Although the results suggest similar progression of cardiac parameters in both treatment arms, a role for revusiran cannot be excluded.

**Clinical Trial Registration:**

NCT02319005.

**Electronic supplementary material:**

The online version of this article (10.1007/s10557-019-06919-4) contains supplementary material, which is available to authorized users.

## Introduction

Transthyretin-mediated amyloidosis (ATTR amyloidosis) is a rapidly progressing, life-threatening disease caused by misfolded transthyretin (TTR) protein that deposits as amyloid fibrils in multiple organs [1–3]. In hereditary TTR-mediated amyloidosis (hATTR amyloidosis), pathogenic mutations in the *TTR* gene cause abnormal amyloid proteins to accumulate in tissues including nerves, heart, and gastrointestinal tract, resulting in a multisystem disease with a heterogeneous clinical presentation [1, 4–6]. Indeed, the majority of patients with hATTR amyloidosis exhibit a mixed phenotype that includes cardiomyopathy and polyneuropathy [7–10]. Wild-type (wt) ATTR-mediated amyloidosis is a non-hereditary type of ATTR amyloidosis, with predominant manifestations of cardiomyopathy and heart failure (HF) [11].

In patients with ATTR amyloidosis with cardiomyopathy, TTR amyloid infiltrates the myocardium leading to heart wall thickening that impairs both diastolic and systolic function [12]. These patients typically present clinically with progressive symptoms of HF and cardiac arrhythmias, most commonly atrial fibrillation [11–13]. Worsening cardiac function is also reflected in increases in cardiac biomarkers and echocardiographic parameters including longitudinal strain, impairment in ambulatory function, and reduced quality of life [12, 14]. The presence of cardiac disease in hATTR amyloidosis is associated with poor outcomes, with a median survival of 3.4 years after diagnosis [15–18]. Death usually occurs from progressive HF [19].

Revusiran is an RNA interference (RNAi) investigational therapeutic that was in clinical development for the treatment of hATTR amyloidosis with cardiomyopathy [20]. The drug comprises a small interfering RNA (siRNA) directed against a region of the human *TTR* mRNA common to wt and documented genetic variants, conjugated to a triantennary *N*-acetylgalactosamine (GalNAc) ligand that delivers it to the liver, the primary site of TTR production [20, 21]. Revusiran is a first-generation GalNAc conjugate based on standard template chemistry. Owing to metabolic stability limitations, revusiran was dosed weekly at relatively high dosages.

Revusiran was previously studied in a Phase 1 study in healthy volunteers [20] and a Phase 2 study with an open-label extension (OLE) in patients with ATTR amyloidosis with cardiomyopathy [22]. The Phase 3 ENDEAVOUR study was a multicenter, randomized, placebo-controlled, double-blind study designed to evaluate the efficacy and safety of revusiran in patients with hATTR amyloidosis with cardiomyopathy. During the study, the Sponsor requested that the independent data-monitoring committee (DMC) for the ENDEAVOUR study assess the benefit–risk profile of revusiran based on investigators’ concerns about new-onset or worsening peripheral neuropathy in some participants in the concurrently running Phase 2 OLE [23]. Although the DMC did not identify any concerns with regard to peripheral neuropathy in the ENDEAVOUR study, an imbalance in mortality in the revusiran arm compared with placebo was observed. As a result, the Sponsor made the decision to discontinue revusiran dosing in all ongoing revusiran studies including the Phase 2 OLE. Here, we present data from the terminated ENDEAVOUR study.

## Methods

### Study Oversight

This study was approved by central and local institutional review boards or ethics committees and performed in accordance with the principles of the Declaration of Helsinki and the International Conference on Harmonization of Technical Requirements for Registration of Pharmaceuticals for Human Use. Written informed consent was obtained from all participating patients. A DMC, comprising a cardiologist, hepatologist, and statistician, reviewed all pertinent benefit–risk data and an independent clinical adjudication committee performed blinded adjudication of the causes of hospitalization and death.

### Study Design

ENDEAVOUR was a multicenter, international, randomized, double-blind, placebo-controlled Phase 3 study carried out at 47 sites in 9 countries (UK, France, Sweden, Spain, Italy, Germany, USA, Belgium, Canada) between December 2014 and March 2017 (NCT02319005). Patients were randomized (2:1) to receive subcutaneous revusiran (500 mg) or placebo (normal saline 0.9% for subcutaneous administration) daily for 5 days during the first week, a dose on Day 7, and a once weekly dose for the remaining study duration. Treatment groups were stratified at randomization for New York Heart Association (NYHA) HF classification (I and II vs. III), *TTR* mutation (V122I versus other *TTR* mutations), and 6-min walk test distance (6MWT) (≤ 325 m vs. > 325 m).

### Patients

Eligible patients were aged 18–90 years, with a documented *TTR* mutation and amyloid deposits confirmed by Congo red (or equivalent) staining or by ^99m-^technetium scintigraphy (Grade 2 or 3 cardiac uptake; centrally confirmed). In patients with monoclonal gammopathy, TTR deposition was required to be confirmed by immunohistochemistry or mass spectrometry.

Patients had a medical history of HF with at least 1 prior hospitalization for HF or clinical evidence of HF that required or was requiring diuretic treatment or was associated with elevated cardiac biomarkers (brain natriuretic peptide [BNP] > 100 pg/ml or *N*-terminal prohormone of BNP [NT-proBNP] > 400 pg/ml). Further eligibility criteria included evidence of cardiac involvement by echocardiogram (interventricular septum thickness ≥ 12 mm) or endomyocardial biopsy demonstrating amyloid deposition, Karnofsky performance status ≥ 50%, 6MWT ≥ 150 m, polyneuropathy disability score < 3, aspartate transaminase (AST) and alanine transaminase (ALT) ≤ 2.0 × upper limit of normal (ULN), albumin > 3 g/dl and total bilirubin < 2.0 mg/dl, and estimated glomerular filtration rate (eGFR) ≥ 30 ml/min/1.73 m^2^. Patients with cardiomyopathy not related to hATTR amyloidosis, NYHA class IV, uncontrolled hypertension, or ischemic heart disease were excluded, as were patients with prior or planned heart or liver transplant during the study. Patients taking TTR stabilizers completed a 14-day wash-out prior to the start of study drug administration.

### Changes to Study Design

During the study, a review of unblinded data by an independent DMC observed an imbalance in mortality in the revusiran arm compared with placebo. Dosing was discontinued and the study was terminated; all patients remaining on-study at the time of study termination were asked to consent to a safety follow-up period. The safety follow-up period included a modified early termination (mET) visit and 2 follow-up visits approximately 30 and 90 days after the mET visit. The second follow-up visit was considered the end of study visit. Assessments carried out in these visits are summarized in Supplementary Fig. 1. Venous lactate and pyruvate measurements from local laboratories were collected only during the mET and subsequent follow-up visits. For purposes of the analyses described in this manuscript, mortality and hospitalization data were considered safety endpoints.

### Study Endpoints

The co-primary efficacy endpoints were change in 6MWT (m) from baseline to 18 months and the percentage reduction in serum TTR levels over 18 months. Cardiovascular (CV) mortality, CV hospitalization, and all-cause mortality were originally planned as secondary efficacy endpoints, but have been moved to safety endpoints as noted above. Echocardiographic parameters, cardiac biomarkers (troponin I and T and NT-proBNP), HF hospitalization, and ^99m-^technetium scintigraphy and cardiac magnetic resonance (CMR) imaging were collected as exploratory efficacy endpoints. Not all planned efficacy endpoints were analyzed due to limited data as a result of early study termination.

### Safety Assessments

Safety assessments, including adverse events (AEs), clinical laboratory testing, urinalysis, 12-lead electrocardiograms, vital signs, physical examination, and antidrug antibodies, were evaluated throughout the study. AEs were coded according to the Medical Dictionary for Regulatory Activities version 17.1. The analysis of safety included all events throughout the study including the safety follow-up period.

Hospitalization and mortality events were collected throughout the treatment and follow-up period. A blinded, independent adjudication committee classified whether all mortality and hospitalization events through the time of database lock were of CV or non-CV origin according to a prespecified charter.

### Pharmacokinetic Assessments

Based on previous studies, the plasma concentration of revusiran reached maximum concentration (C_max_) at approximately 2.5 h (T_max_) after subcutaneous injection [20]. Accordingly, revusiran plasma levels were measured 2.5 h ± 1 h post dose on Day 0, Month 6, and Month 12 using a validated liquid chromatography, high-resolution accurate-mass mass spectrometry method.

### Pharmacodynamic Assessments

Serum TTR levels were measured at baseline, and at Months 1, 2, 3, 6, 9, 12, and 15. Total serum TTR was quantified using a custom-developed, validated, sandwich enzyme-linked immunosorbent assay.

### Efficacy Assessments

6MWT was assessed at baseline and at 3, 6, 12, and 18 months. Cardiac structure and function were assessed by echocardiogram with Doppler at baseline and every 6 months thereafter. Echocardiograms were obtained at the study sites according to a prespecified protocol and underwent blinded assessment in a cardiac imaging core laboratory. ^99m-^Technetium scintigraphy and CMR with late gadolinium enhancement were also obtained at selected sites in a subset of patients. CMR was obtained from all patients except those with contraindications (i.e., pacemakers, defibrillators, inadequate renal function, or gadolinium allergy).

Cardiac biomarkers were analyzed at a central laboratory: serum NT-proBNP and troponin T by electrochemiluminescence immunoassay (Roche Diagnostics, Rotkreuz, Switzerland); troponin I by chemiluminescence assay (Siemens Healthineers, Erlangen, Germany).

### Statistical Analysis

The modified intent-to-treat (mITT) population, defined as all randomized patients who received at least 1 dose of study drug or placebo, was used for efficacy analyses. The safety population comprised all patients who received at least 1 dose of study drug or placebo (analyzed as treated) and was used for safety analyses. As there were no differences in randomization and treatment with respect to study drug administration, the mITT and safety populations were the same.

Planned statistical tests and subgroup analyses for co-primary, secondary, and exploratory endpoints could not be performed due to premature discontinuation of dosing and study termination. As a result, hypothesis-generating analyses were performed to examine changes between treatment arms for co-primary parameters (6MWT and TTR), key secondary parameters (death and hospitalizations), and key exploratory parameters (echo and cardiac biomarkers). One-way analysis of variance (i.e., Kruskal–Wallis test) was performed to compare distributions of C_max_ between groups (died versus alive) at each visit and across 3 renal function groups.

Descriptive statistics were generated for any continuous data. Time from first dose to first event (mortality and/or hospitalization) analyses used the Kaplan–Meier method. These event analyses were summarized for 2 time periods, on-treatment and on-study. The on-treatment period was defined as the time from first dose of study drug to November 4, 2016 (approximately 30 days after notification to the study sites to discontinue dosing). The on-study period was defined as the time from the first dose of study drug through the latest day on-study. Exposure-adjusted mortality and/or hospitalization rates were calculated as total number of events divided by total person-years of exposure. Descriptive statistics of key cardiac and echocardiogram parameters were examined in a subset of patients with complete (non-missing) data at baseline and each post-baseline visit up to and including Month 6 to understand trends over time. To further understand factors associated with mortality, patients were compared by outcome (died versus alive) within the revusiran arm. Comparisons of baseline disease characteristics and trends in cardiac biomarkers and echo parameters using all data until Month 6 were also examined. In addition, TTR knockdown over time was compared by outcome within the revusiran arm. Plasma revusiran C_max_ was also summarized by outcome (died or alive while on-treatment), visit (baseline, Month 6, and Month 12), and renal function (eGFR: 30 to < 60, 60 to < 90, and ≥ 90 ml/min/1.73 m^2^).

## Results

### Patient Demographics and Disposition

The study was fully enrolled prior to discontinuation. All 206 patients who were enrolled and randomized in the study (revusiran: *n* = 140; placebo: *n* = 66) received at least 1 dose of study drug (Supplementary Fig. 2). Overall, 115 patients in the revusiran arm and 64 patients in the placebo arm were on-study at the time of dosing discontinuation. The mET visit at the end of the safety follow-up period was completed by 92 patients in the revusiran arm and 51 patients in the placebo arm. Owing to the early study termination, the median duration of revusiran treatment was 6.71 months (range 2.11–16.32) and median number of doses of revusiran received was 33.5 doses (range 13–76). There were 36 (25.7%) patients with ≥ 9 months and 16 (11.4%) patients with ≥ 12 months of exposure to revusiran.

Most patients were male and either white or black/African American with a median age of 69 years. Demographics were generally balanced between treatment arms. While age was balanced between treatment arms, 30.7% of patients treated with revusiran were age ≥ 75 years compared with 18.2% of patients on the placebo arm (Table 1).

**Table 1:** Baseline demographics, disease characteristics, key cardiac biomarkers, and echocardiogram parameters of study population and exposure to study drug

	Placebo	Revusiran	Total
	(*n* = 66)	(*n* = 140)	(*n* = 206)
Demographics			
Age at randomization, years			
Median (range)	68.0 (38–81)	69.0 (37–86)	69.0 (37–86)
Age category, *n* (%)			
18–64	25 (37.9)	41 (29.3)	66 (32.0)
65–74	29 (43.9)	56 (40.0)	85 (41.3)
≥ 75	12 (18.2)	43 (30.7)	55 (26.7)
Sex, *n* (%)			
Male	53 (80.3)	105 (75.0)	158 (76.7)
Race, *n* (%)			
White	29 (43.9)	66 (47.1)	95 (46.1)
Black	36 (54.5)	68 (48.6)	104 (50.5)
American Indian or Alaska Native	1 (1.5)	0	1 (0.5)
Other	0	6 (4.3)	6 (2.9)
Geographic region, *n* (%)			
North America	45 (68.2)	96 (68.6)	141 (68.4)
Western Europe	21 (31.8)	44 (31.4)	65 (31.6)
Mean (SD) mBMI^*^	1085.4 (196.8)	1113.7 (253.5)	1104.6 (236.6)
Disease characteristics			
* TTR* mutation, *n* (%)			
Val122Ile	37 (56.1)	80 (57.1)	117 (56.8)
Thr60Ala	12 (18.2)	21 (15.0)	33 (16.0)
Glu89Gln	2 (3.0)	3 (2.1)	5 (2.4)
Other	15 (22.7)	36 (25.7)	51 (24.8)
PND score, *n* (%)			
0	35 (53.0)	62 (44.3)	97 (47.1)
1	20 (30.3)	55 (39.3)	75 (36.4)
2	11 (16.7)	23 (16.4)	34 (16.5)
NYHA class, *n* (%)			
I	4 (6.1)	13 (9.3)	17 (8.3)
II	42 (63.6)	83 (59.3)	125 (60.7)
III	20 (30.3)	44 (31.4)	64 (31.1)
KCCQ Overall Summary Score (SD)	65 (22.1)	67 (20.0)	67 (22.0)
Mean (SD) 6MWT at baseline, m	400 (131.3)	376 (117.6)	383.6 (122.4)
Mean (SD) time from diagnosis to date of first dose, months^†^	12 (12.4)	15 (28.7)	14 (24.7)
Renal impairment, *n* (%)			
Normal: eGFR ≥ 90 ml/min/1.73 m^2^	7 (10.6)	15 (10.7)	22 (10.7)
Mild: eGFR ≥ 60 to < 90 ml/min/1.73 m^2^	28 (42.4)	63 (45.0)	91 (44.2)
Moderate: eGFR ≥ 30 to < 60 ml/min/1.73 m^2^	31 (47.0)	62 (44.3)	93 (45.1)
Medical history of peripheral neuropathy, *n* (%)^‡^	15 (22.7)	36 (25.7)	51 (24.8)
Key cardiac biomarkers and echocardiogram parameters			
Median (range) troponin I, μg/l	0.13 (0–0.95)	0.12 (0–1.66)	0.13 (0–1.66)
Median (range) NT-proBNP, pg/ml	2719 (51–16,170)	2371 (74–32,470)	2511 (51–32,470)
Mean (SD) intraventricular septum thickness, mm	18.6 (2.5)	18.2 (2.6)	18.3 (2.6)
Mean (SD) average peak longitudinal strain, %	−10.4 (3.6)	−11.0 (3.4)	−10.8 (3.5)
Mean (SD) left ventricular ejection fraction, %	52.2 (10.3)	53.1 (12.0)	52.8 (11.5)
Mayo risk staging^§^			
High	15 (23)	36 (26)	51 (25)
Intermediate	19 (29)	37 (26)	56 (27)
Low	32 (48)	67 (48)	99 (48)
Median (range) exposure to study drug^‖^	7.7 (2.1–16.4)	6.7 (2.1–16.3)	–
Median (range) doses received	37.5 (14–76)	33.5 (13–76)	–

Percentages are based on the number of patients randomized. Baseline was defined as the last value of the parameter prior to the first dose date

^*^mBMI was calculated as the product of BMI (kg/m^2^) and albumin (g/l).^†^Calculated as (date of first dose − date of diagnosis +1)/30.4.^‡^Based on standardized MedDRA HLT for peripheral neuropathy NEC.^§^Risk groups [24]: High risk – Both biomarkers above threshold at baseline; Intermediate risk – 1 above threshold at baseline; Low risk – Neither above at baseline. Biomarker thresholds – Troponin T > 0.05 ng/ml, NT-proBNP > 3000 pg/ml^‖^Duration of exposure in months was calculated as (the date of last dose of study drug – the date of the first dose of study drug +1)/30.4

*6MWT* 6-min walk test distance, *eGFR* estimated glomerular filtration rate, *HLT* high-level term, *KCCQ* Kansas City Cardiomyopathy Questionnaire, *mBMI* modified body mass index, *NYHA* New York Heart Association heart failure classification, *MedDRA* Medical Dictionary for Regulatory Affairs, *NEC* not elsewhere classified, *NT-proBNP N*-terminal prohormone of brain natriuretic peptide, *PND* polyneuropathy disability, *SD* standard deviation

Baseline disease characteristics were balanced between treatment arms and demonstrated significant clinical HF. Cardiac amyloidosis severity, distribution of ATTR mutations, and renal function were well balanced between arms. All patients had a history of HF with 8.3%, 60.7%, and 31.1% of patients NYHA classification I, II, or III, respectively. Overall, 24.8% of patients reported medical history of peripheral neuropathy (Table 1). Median (range) troponin I levels were 0.13 μg/l (0–1.66), and median (range) NT-proBNP levels were 2511 pg/ml (51–32,470). Mean ± standard deviation intraventricular septum wall thickness was 18.3 ± 2.6 mm, average peak longitudinal strain was −10.8 ± 3.5%, and left ventricular ejection fraction was 52.8 ± 11.5%.

### Safety and Tolerability

**Fig. 1:**
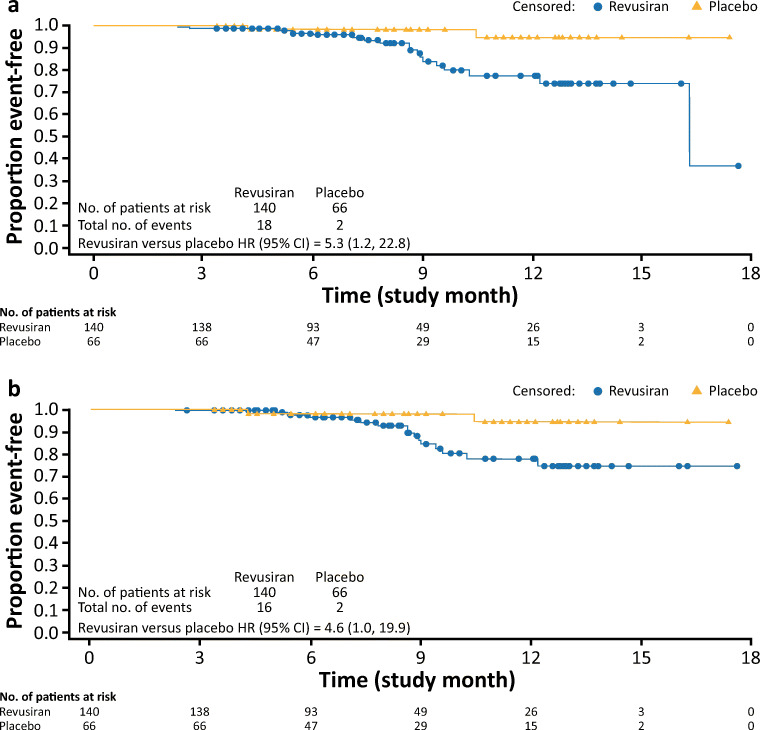
All-cause and cardiovascular mortality (modified intent-to-treat population). (**a**) Time to all-cause mortality. (**b**) Time to cardiovascular mortality. CI = confidence interval; HR = hazard ratio

As noted above, the study was prematurely discontinued due to an imbalance of deaths observed in the revusiran group (18 patients, 12.9%) compared with the placebo group (2 patients, 3.0%) during the on-treatment period (Table 2 and Fig. 1). The majority of deaths in both groups were CV events, and most were categorized as HF (Supplementary Table 1). In addition, there were 2 CV deaths categorized as sudden cardiac death, with 1 occurring in each treatment arm. Over the course of the study, including the safety follow-up period, deaths were reported in 23 patients (16.4%) in the revusiran group and 7 patients (10.6%) in the placebo group (Table 3 and Supplementary Fig. 3). Three of the deaths in the revusiran group (2 due to cardiac failure and 1 due to congestive cardiac failure) were considered possibly related to the study drug by the investigator. The exposure-adjusted CV mortality rates were 0.168 and 0.042 per person-year in the revusiran and placebo arms, respectively.

**Table 2:** Summary of mortality or first hospitalization events from first dose of study drug while on-treatment^*^

Event	Placebo	Revusiran	Hazard ratio (95% CI)
	(*n* = 66)	(*n* = 140)	(revusiran versus placebo)^†^
	*n* (%)	*n* (%)	
All-cause mortality	2 (3.0)	18 (12.9)	5.3 (1.2, 22.8)
CV mortality^‡^	2 (3.0)	16 (11.4)	4.6 (1.0, 19.9)
All-cause hospitalization^§^	24 (36.4)	67 (47.9)	1.4 (0.9, 2.2)
CV hospitalization	21 (31.8)	49 (35.0)	1.1 (0.7, 1.8)
HF hospitalization	13 (19.7)	41 (29.3)	1.6 (0.8, 2.9)

^*^On-treatment events classified as all events that occurred on or prior to November 4, 2016.^†^The hazard ratio with associated 95% CI is based on the Cox proportional hazard models for time to events with randomized treatment arm as a covariate.^‡^Deaths observed were adjudicated as heart failure or sudden cardiac death and did not include any vascular events (e.g., stroke, AMI, or CV hemorrhage). ^§^All-cause hospitalization events occurring on-treatment include any all-cause hospitalization events (CV and non-CV)

*AMI* acute myocardial infarction, *CI* confidence interval, *CV* cardiovascular, *HF* heart failure

**Table 3:** Overview of safety during the study, including safety follow-up period

	Placebo (*n* = 66)	Revusiran (*n* = 140)
	Number of patients (%)	
Event		
Any AE	62 (93.9)	136 (97.1)
Any severe AE	19 (28.8)	55 (39.3)
Any SAE	34 (51.5)	83 (59.3)
Any AE leading to discontinuation of trial regimen	1 (1.5)	20 (14.3)
Any AE leading to withdrawal from the trial	0	9 (6.4)
Death	7 (10.6)	23 (16.4)
SAEs occurring in ≥ 5% patients in either treatment arm		
Cardiac failure	9 (13.6)	25 (17.9)
Cardiac failure acute	9 (13.6)	15 (10.7)
Cardiac failure congestive	4 (6.1)	9 (6.4)
Atrial fibrillation	2 (3.0)	7 (5.0)
Neuropathy peripheral	0	7 (5.0)
Atrial flutter	4 (6.1)	2 (1.4)
AEs occurring in ≥ 15% patients in either treatment arm		
Cardiac failure	12 (18.2)	31 (22.1)
Cough	10 (15.2)	25 (17.9)
Neuropathy peripheral	6 (9.1)	25 (17.9)
Edema peripheral	12 (18.2)	25 (17.9)
Injection site pain	4 (6.1)	23 (16.4)
Constipation	11 (16.7)	21 (15.0)
Dizziness	13 (19.7)	18 (12.9)
Safety areas of interest		
Cardiac events^*^	36 (54.5)	82 (58.6)
Severe cardiac events	15 (22.7)	35 (25.0)
Serious cardiac events	25 (37.9)	56 (40.0)
Hepatic events^†^	9 (13.6)	48 (34.3)
Severe hepatic events	0	7 (5.0)
Serious hepatic events	0	8 (5.7)
Renal events^‡^	7 (10.6)	31 (22.1)
Severe renal events	3 (4.5)	6 (4.3)
Serious renal events	3 (4.5)	6 (4.3)
Peripheral neuropathy events^§^	8 (12.1)	28 (20.0)
Severe peripheral neuropathy events	1 (1.5)	3 (2.1)
Serious peripheral neuropathy events	0	7 (5.0)
ISR events^‖^	7 (10.6)	54 (38.6)
Severe ISRs	0	0
Serious ISRs	0	0
Myopathy events^**^	5 (7.6)	4 (2.9)
Severe myopathy events	0	0
Serious myopathy events	0	1 (0.7)
Lactic acidosis events^††^	4 (6.1)	15 (10.7)
Severe lactic acidosis events	0	0
Serious lactic acidosis events	0	1 (0.7)

^*^Cardiac events include AEs mapping to the SOC “cardiac disorders”.^†^Hepatic events include AEs mapping to the SMQ “drug-related hepatic disorders”.^‡^Renal events include AEs mapping to the SMQ “acute renal failure”.^§^Peripheral neuropathy events include AEs mapping to the HLT “peripheral neuropathy”.^‖^Injection site reaction events include AEs mapping to the HLT “injection site reaction”.^**^Myopathy events include AEs mapping to the SMQ “myopathy” (narrow terms) and additional PTs of “biopsy muscle abnormal”, “electromyogram abnormal”, “muscle disorder”, and “muscular weakness”.^††^Lactic acidosis events include AEs mapping to the SMQ “lactic acidosis” (e.g., “blood lactate increased”, “lactic acidosis”, “blood bicarbonate decreased”)

*AE* adverse event, *HLT* high-level term, *ISR* injection site reaction, *PT* preferred term, *SAE* serious adverse event, *SMQ* standardized Medical Dictionary for Regulatory Affairs query, *SOC* system organ class

To understand factors associated with mortality, additional analyses were performed to compare patients in the revusiran arm who died and those who were alive at the end of the on-treatment period. Demographic and disease characteristics at baseline showed that a higher percentage of patients in the revusiran arm who died on-treatment were ≥ 75 years of age, were categorized as NYHA class III, had a shorter mean 6MWT distance, had lower mean eGFR, had lower mean cardiac output, and had higher median NT-proBNP and troponin I than those patients who were alive throughout the on-treatment period (Table 4). Mean values of cardiac biomarkers and echocardiographic assessment indicated more abnormal values at baseline in patients who died versus patients who were alive. In patients who died, NT-proBNP and global longitudinal strain showed more worsening through Month 6 compared with patients who were alive (Table 5).

**Table 4:** Summary of baseline demographics, disease characteristics, key cardiac biomarkers, and echocardiogram parameters in the revusiran arm by outcome (modified intent-to-treat population)

	Patients in the revusiran arm alive on-treatment	Patients in the revusiran arm who died on-treatment
	(*n* = 122)	(*n* = 18)
Demographics		
Age at randomization, years		
Median (range)	68.0 (37–86)	76.5 (56–82)
Age category, *n* (%)		
18–64	39 (32.0)	2 (11.1)
65–74	50 (41.0)	6 (33.3)
≥ 75	33 (27.0)	10 (55.6)
Sex, *n* (%)		
Male	96 (78.7)	9 (50.0)
Race, *n* (%)		
White	58 (47.5)	8 (44.4)
Black	60 (49.2)	8 (44.4)
Other	4 (3.3)	2 (11.1)
Ethnicity, *n* (%)		
Hispanic or Latino	2 (1.6)	3 (16.7)
Not Hispanic or Latino	114 (93.4)	15 (83.3)
Not reported/unknown	6 (4.9)	0
Mean (SD) mBMI^*^	1132.4 (248.1)	987.7 (259.9)
Disease characteristics		
* TTR* mutation, *n* (%)		
Val122Ile	67 (54.9)	13 (72.2)
Thr60Ala	19 (15.6)	2 (11.1)
Glu89Gln	3 (2.5)	0
Other	33 (27.0)	3 (16.7)
NYHA class, *n* (%)		
I	13 (10.7)	0
II	77 (63.1)	6 (33.3)
III	32 (26.2)	12 (66.7)
Mean (SD) 6MWT at baseline, m	385.9 (115.2)	309.1 (114.7)
Renal impairment, *n* (%)		
Normal: eGFR ≥ 90 ml/min/1.73 m^2^	15 (12.3)	0
Mild: eGFR > 60 to < 90 ml/min/1.73 m^2^	57 (46.7)	6 (33.3)
Moderate: eGFR > 30 to < 60 ml/min/1.73 m^2^	50 (41.0)	12 (66.7)
Mean (SD) time from diagnosis to date of first dose, months^†^	15.2 (30.4)	10.5 (10.0)
Key cardiac biomarkers and echocardiogram parameters		
Median (range) troponin I, μg/l	0.11 (0–1.66)	0.20 (0.08–0.56)
Median (range) NT-proBNP, pg/ml	2254 (74–32,470)	3547 (1412–18,020)
Mean (SD) intraventricular septum thickness, mm	18.2 (2.6)	18.2 (2.0)
Mean (SD) average peak longitudinal strain, %	−11.2 (3.4)	−9.1 (3.1)
Mean (SD) left ventricular ejection fraction, %	53.5 (12.1)	50.6 (11.3)
Mean (SD) cardiac output, l/min	3.5 (1.1)	2.7 (1.1)

Percentages are based on the number of patients randomized. Baseline was defined as the last value of the parameter prior to the first dose date

^*^mBMI was calculated as the product of BMI (kg/m^2^) and albumin (g/l)^.†^Calculated as (date of first dose − date of diagnosis +1)/30.4

*6MWT* 6-min walk test distance, *eGFR* estimated glomerular filtration rate, *mBMI* modified body mass index, *NYHA* New York Heart Association heart failure classification, *NT-proBNP N*-terminal prohormone of brain natriuretic peptide, *SD* standard deviation, *TTR* transthyretin

**Table 5:** Summary of cardiac biomarker and echocardiogram parameters by outcome during the on-treatment period (safety population)

	Patients in the revusiran arm alive on-treatment	Patients in the revusiran arm who died on-treatment
Mean troponin I (SD), μg/l		
Baseline	*n* = 122	*n* = 18
	0.17 (0.209)	0.24 (0.140)
Month 3	*n* = 107	*n* = 13
	0.16 (0.201)	0.16 (0.093)
Month 6	*n* = 66	*n* = 11
	0.22 (0.462)	0.17 (0.066)
Mean NT-proBNP (SD), pg/ml		
Baseline	*n* = 117	*n* = 17
	3212 (3991.7)	6022 (5030.1)
Month 3	*n* = 105	*n* = 13
	3316 (5297.9)	4466 (3403.4)
Month 6	*n* = 66	*n* = 12
	3066 (2471.4)	7086 (3688.1)
Mean (SD) LVEF, %		
Baseline	*n* = 117	*n* = 18
	53.5 (12.1)	50.6 (11.3)
Month 6	*n* = 65	*n* = 10
	55.1 (11.7)	53.7 (9.5)
Mean (SD) GLS, %		
Baseline	*n* = 115	*n* = 16
	−11.2 (3.4)	−9.1 (3.1)
Month 6	*n* = 65	*n* = 11
	−11.4 (3.7)	−8.2 (3.1)

Patients were classified into “died” group if they died on-treatment. For each post-baseline visit interval, the label of the visit was used

*NT-proBNP N*-terminal prohormone of brain natriuretic peptide, *LVEF* left ventricular ejection fraction, *GLS* global longitudinal strain, *SD* standard deviation

**Fig. 2:**
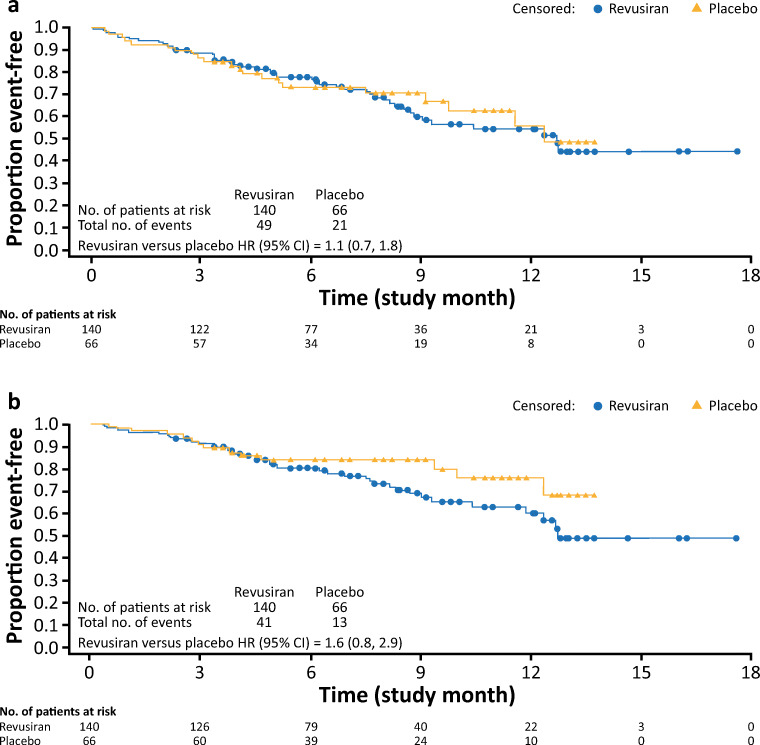
Time to all-cause, cardiovascular, and heart failure hospitalization (modified intent-to-treat population). (**a**) Time to first cardiovascular hospitalization. (**b**) Time to first heart failure hospitalization. CI = confidence interval; HR = hazard ratio

Data on CV and all-cause mortality and hospitalizations are shown in Table 2. When cardiac serious AEs (SAEs) that resulted in hospitalization were compared during the on-treatment period, both the proportion of patients who reported at least 1 CV hospitalization (revusiran: 35.0%; placebo: 31.8%) (Table 2) and the median time to first CV hospitalization (revusiran: 12.7 months [95% confidence interval (CI) 8.8, not reached]; placebo: 12.4 months [95% CI 9.8, not reached]) (Fig. 2) were similar between the 2 treatment groups (hazard ratio [95% CI] 1.1 [0.7, 1.8]). The majority of CV hospitalizations on-treatment in both groups were categorized as HF (revusiran: 41 of 49 events [83.7%]; placebo: 13 of 21 events [61.9%]). Time to first HF hospitalization by treatment group demonstrated a trend similar to that observed for CV hospitalizations, with a hazard ratio (95% CI) of 1.6 (0.8, 2.9) (Table 2 and Fig. 2). Exposure-adjusted CV hospitalization rates per person-years were 0.786 in the revusiran group and 0.797 in the placebo group. The rate of all-cause hospitalization was 47.9% in the revusiran group and 36.4% in the placebo group (hazard ratio [95% CI] 1.4 [0.9, 2.2]).

The composite analysis of potentially competing clinical events, time to CV mortality, or first HF hospitalization on-treatment had a hazard ratio (95% CI) of 1.5 (0.8, 2.7) (Supplementary Fig. 4).

During the study, including the safety follow-up period (Table 3), 97.1% of patients in the revusiran group and 93.9% of patients in the placebo group reported AEs. A higher proportion of patients in the revusiran group compared with placebo reported severe AEs (39.3% and 28.8%, respectively), SAEs (59.3% and 51.5%), and AEs that led to discontinuation of treatment (14.3% and 1.5%) or withdrawal from the trial (6.4% and 0%). In both treatment groups, SAEs of cardiac failure and cardiac failure acute were reported in ≥ 10% of patients (Table 3). AEs that led to discontinuation of revusiran in ≥ 2 patients were cardiac failure (3.6%) and cardiac failure acute, cardiogenic shock, and cachexia (1.4% each). The frequency of cardiac SAEs (40.0% and 37.9% in revusiran and placebo groups, respectively) and cardiac AEs (58.6% and 54.5%) were balanced between the revusiran and placebo arms (Table 3) with the AEs in both arms being similar in nature.

As patients with HF often have concomitant hepatic or renal impairment, additional analyses assessed hepatic and renal events. More patients in the revusiran group (34.3%) had hepatic events than in the placebo group (13.6%) (Table 3). Of these, the majority of patients had hepatic events that corresponded to laboratory abnormalities and were considered mild or moderate in severity. Seven patients (5.0%) in the revusiran group had severe hepatic events compared with none in the placebo group. Additionally, 4 patients (2.9%) in the revusiran group had elevations of ALT or AST ≥ 3 times the ULN with accompanying increases in total bilirubin > 2 times the ULN. All of these patients had medical conditions or factors which contributed to the hepatic events and/or transaminase elevations including concomitant worsening of end-stage HF, multisystem organ failure in the setting of an infected pleural effusion and cardiac cachexia, metastatic cholangiocarcinoma, and cholestatic hepatitis in 1 patient with history of heavy alcohol use and long-term treatment with azithromycin.

Similarly, more patients in the revusiran group (22.1%) had renal events than in placebo (10.6%) (Table 3). The majority of patients had renal events that were considered mild or moderate in severity. Serious renal events were reported in 6 (4.3%) patients in the revusiran group and 3 (4.5%) patients in the placebo group. In both groups, the patients had medical conditions or factors that contributed to these events, including concomitant worsening of end-stage HF, multisystem organ failure with infected pleural effusion and cardiac cachexia, concurrent hypotension or hypovolemia, and acute diverticulitis with diarrhea and volume overload.

Peripheral neuropathy events (20.0% and 12.1%, in revusiran and placebo arms, respectively) and serious peripheral neuropathy events (5.0% and 0%) were reported more frequently in the revusiran group than the placebo group (Table 3). Most patients in both groups had events that were mild or moderate in severity. Three (2.1%) patients in the revusiran group and 1 (1.5%) patient in the placebo group had peripheral neuropathy events that were considered severe. Events of myopathy were reported in 2.9% and 7.6% in the revusiran and placebo arms, respectively, and lactic acidosis events were reported in 10.7% and 6.1% in the revusiran and placebo arms, respectively. All events were mild or moderate in severity. Blood lactate levels were only measured during the follow-up period and showed elevations in both arms, with most elevations being < 2 × ULN (Supplementary Fig. 5). Throughout the study and safety follow-up period, measurements of anion gap were similar between the revusiran and placebo groups (data not shown).

### Pharmacokinetics

Mean plasma revusiran C_max_ increased slightly from baseline to Month 6 and appeared to have achieved steady state by Month 6 with no further increase at Month 12 (Supplementary Table 2). Mean C_max_ values appeared to be similar in patients who died and those who were alive during treatment considering the large overlap of the standard deviations at both baseline and at Month 6 (*p* > 0.13).

There were no apparent differences in revusiran C_max_ between patients with mild (eGFR: 30 and < 60 ml/min/1.73 m^2^) or moderate (eGFR: 60 to < 90 ml/min/1.73 m^2^) renal impairment when compared with patients with normal (eGFR: ≥ 90 ml/min/1.73 m^2^) renal function at Weeks 0, 26, and 52 (*p* > 0.20) (Supplementary Fig. 6). It is important to note the high degree of interpatient variability, the small numbers of patients with normal renal function at all time points, and the small number of patients with C_max_ and eGFR data at Month 12.

### Pharmacodynamics

Revusiran resulted in a mean > 80% reduction of serum TTR which was apparent from Month 1 and maintained through Month 15 (Supplementary Fig. 7). The mean maximum ± standard error of the mean (SEM) serum TTR reduction was 89.5 ± 0.589% relative to baseline. Values of mean maximum TTR knockdown (92.0% and 89.1%) were similar in patients who died and those who were alive. Following discontinuation of dosing, mean serum TTR levels returned to near baseline within 90 days.

### Efficacy

Given the limited duration of exposure due to early termination of the study only descriptive analyses of key efficacy parameters are presented.

In both treatment arms, patients declined in 6MWT at 6 months compared with baseline. The mean change from baseline in 6MWT at 6 months was similar with revusiran (−21.4 m; SEM: 9.0; *n* = 76) and placebo (−17.6 m; SEM: 11.8; *n* = 41).

Change over time from baseline to Month 6 in key echocardiogram parameters and cardiac biomarker data were similar between treatment arms and showed no clinically meaningful improvement in the revusiran arm compared with placebo (Supplementary Table 3).

Analyses of the secondary endpoints of death and hospitalizations are described above in safety results. Planned statistical tests could not be performed for ^99m-^technetium scintigraphy and CMR imaging due to limited data.

## Discussion

The Phase 3 ENDEAVOUR study was designed to investigate the effect of revusiran, a first-generation GalNAc–siRNA conjugate targeting TTR, in patients with hATTR amyloidosis with cardiomyopathy, a debilitating condition with an average life expectancy of a median 3.4 years from diagnosis. The study population comprised predominantly older patients with advanced clinical HF. Dosing in the trial was stopped after a median follow-up of 6.71 months in patients treated with revusiran due to an imbalance in mortality observed between the treatment arms. The deaths on-study were predominantly CV due to HF, consistent with the natural history of the disease, and most were considered unrelated to study treatment by the investigator at the time of the event prior to discontinuation of dosing in the trial. An extensive analysis of safety was performed in an effort to understand the cause of the mortality imbalance.

Comparing the placebo-controlled data, baseline demographic and disease characteristics of the 2 treatment arms were balanced, except for a greater proportion of patients over the age of 75 years in the revusiran arm compared with placebo. However, differences in the age distribution do not appear to fully explain the observed difference in the mortality rate. Surprisingly, the imbalance in deaths, primarily CV due to HF, was not paralleled by the expected increase in CV hospitalizations, and key echocardiographic parameters and cardiac biomarkers progressed at a similar rate over time in the 2 treatment arms. With respect to AEs, compared with placebo, patients on revusiran reported an increased incidence of peripheral neuropathy, hepatic events that were primarily laboratory elevations, and renal events (Table 3).

We also analyzed patient characteristics by outcome and found that in the revusiran arm deaths occurred in an at-risk group of patients with baseline clinical, echocardiographic, and biomarker evidence of disease that was more advanced than that of the patients on-treatment who were alive. However, there were similar at-risk patients in the placebo arm. Finally, patients who died had similar revusiran pharmacokinetic exposures and pharmacodynamic responses as those who did not.

A clear causative mechanism could not therefore be identified for the mortality imbalance. Short- and long-term rat and non-human primate chronic toxicology studies (of up to 2 years’ duration) did not reveal any corresponding toxicities to those observed in the ENDEAVOUR study [25] (data on file). However, the combination of an increase in deaths in the revusiran arm and other notable imbalances in AEs (peripheral neuropathy, hepatic, and renal events) suggests that drug-mediated toxicity may have been a factor in the outcome.

In light of the negative ENDEAVOUR outcome, and notwithstanding the similarities in revusiran-mediated TTR reductions between those who died on drug versus those who did not, an important question arises regarding the safety of TTR-lowering approaches in hATTR amyloidosis. However, the safety and efficacy of TTR lowering as a therapeutic strategy have been validated by another therapy that reduces TTR levels using double-stranded RNAi (patisiran) [9], which has recently been approved for the treatment of patients with hATTR amyloidosis with polyneuropathy. The pivotal Phase 3 trial of patisiran (APOLLO) included a prespecified subpopulation of patients with evidence of cardiac involvement, which comprised a majority (56%) of the overall study population [26]. In the APOLLO study, TTR reduction was associated with an acceptable safety profile in both the overall study population [9] and the prespecified cardiac subpopulation [26]. Importantly, the exposure-adjusted mortality rate was lower for patisiran versus placebo, and in post hoc analysis of safety data a reduction in event rates in the patisiran arm compared with placebo was observed for both any hospitalization and/or all-cause death as well as cardiac hospitalizations and/or all-cause death [26]. Collectively, these data suggest the potential benefit of an siRNA targeting TTR for treating cardiac manifestations of this disease; additional studies are, however, needed.

Accordingly, another GalNAc–siRNA conjugate targeting TTR, vutrisiran (ALN-TTRsc02), will be used to further explore the benefit–risk profile of RNAi therapeutics across the full spectrum of ATTR amyloidosis, including patients with hereditary and wt cardiomyopathy. Compared with revusiran, which is a first-generation (standard template chemistry) GalNAc–siRNA conjugate, and thus prone to rapid in vivo nuclease-mediated degradation, vutrisiran is a second-generation compound (enhanced stabilization chemistry [ESC]), with far greater metabolic stability leading to significantly augmented potency and durability [27]. As such, the exposure to revusiran, given at 500-mg weekly doses in the ENDEAVOUR study, was 28 g of siRNA in the first year, whereas vutrisiran achieves the same degree of TTR reduction at 25 mg every 3 months (100 mg annually), equating to a 280-fold lowered drug exposure. For ESC GalNAc–siRNA conjugates, lowered exposures are expected to lead to more favorable safety results [25]. Consistent with this, several ESC GalNAc–siRNA compounds are in, or have recently completed, Phase 3 studies without similar findings to those seen with revusiran. Importantly, this group of ESC GalNAc–siRNA conjugates sharing significant structural and chemical similarities includes inclisiran, administered at 300 mg every 6 months. Inclisiran has shown encouraging safety and efficacy data in early development [28, 29] and is currently approaching the end of Phase 3 studies with the program fully enrolled. The population evaluated in the inclisiran studies is composed of patients with hypercholesterolemia and atherosclerotic CV disease (ASCVD) or ASCVD risk who would be expected to be prone to cardiac events. To date, with over 3000 patients enrolled, with 2750 patient-years of exposure to inclisiran, safety is encouraging [30], which in turn suggests favorable cardiac tolerability for ESC GalNAc–siRNA conjugates.

## Conclusions

Following a thorough investigation, a clear causative mechanism for the mortality imbalance observed between treatment arms on the Phase 3 ENDEAVOUR study could not be identified. However, it is possible that revusiran may have contributed to the finding and further development of this compound has been discontinued. Data from the Phase 3 APOLLO study of patisiran support the therapeutic hypothesis of TTR reduction as a potential approach for treatment of cardiomyopathy in hATTR amyloidosis. Further studies are planned to evaluate the efficacy and safety of both patisiran and ESC siRNA–GalNAc conjugates with enhanced metabolic stability in patients with ATTR cardiac amyloidosis.

## Electronic supplementary material

ESM 1 (DOCX 2.58 mb)
