# Supplementary material for: Phase 3 Multicenter Study of Revusiran in Patients with Hereditary Transthyretin-Mediated (hATTR) Amyloidosis with Cardiomyopathy (ENDEAVOUR)
Source: Cardiovasc Drugs Ther. 2020 Feb 15;34(3):357–70. doi: 10.1007/s10557-019-06919-4 (PMC7242280; doi:10.1007/s10557-019-06919-4)

# Supplementary Material

**Supplementary Table 1** Overview of all deaths during the study

| Patient number | AE(s) leading to death (preferred term) | Adjudicated death category | Duration of treatment (days) | Study day of death |  |
| --- | --- | --- | --- | --- | --- |
| On-treatment deaths (deaths up to November 4, 2016): Revusiran arm | | | | |  |
| Patient 1 | Cardiac arrest | CV (sudden cardiac death) | 64 | 71 |  |
| Patient 2 | Infectious pleural effusion | Non-CV | 66 | 80 |  |
|  | Cachexia | Non-CV |  |  |  |
| Patient 3 | Renal failure acute | CV (heart failure) | 150 | 154 |  |
| Patient 4 | Cardiac failure acute | CV (heart failure) | 141 | 163 |  |
| Patient 5 | Cardiac failure acute | CV (heart failure) | 177 | 181 |  |
| Patient 6 | Cardiac failure congestive | CV (heart failure) | 184 | 216 |  |
| Patient 7 | Cardiac failure chronic | CV (heart failure) | 220 | 224 |  |
| Patient 8 | Cardiogenic shock | CV (heart failure) | 184 | 238 |  |
| Patient 9 | Cardiac failure | CV (heart failure) | 232 | 263 |  |
| Patient 10 | Acute respiratory failure | CV (heart failure) | 157 | 263 |  |
| Patient 11 | Cachexia | CV (heart failure) | 246 | 269 |  |
| Patient 12 | Cardiac failure | CV (heart failure) | 260 | 272 |  |
| Patient 13 | Cardiac failure congestive | CV (heart failure) | 268 | 274 |  |
| Patient 14 | Cardiogenic shock | CV (heart failure) | 275 | 286 |  |
| Patient 15 | Renal failure | CV (heart failure) | 267 | 292 |  |
| Patient 16 | Cardiac failure congestive | CV (heart failure) | 288 | 312 |  |
| Patient 17 | Cardiac failure | CV (heart failure) | 351 | 371 |  |
| Patient 18 | Sepsis | Non-CV | 483 | 495 |  |
|  | Pneumonia | Non-CV |  |  |  |
|  | Subarachnoid hemorrhage | Non-CV |  |  |  |
| On-treatment deaths (deaths up to November 4, 2016): Placebo arm | | | | | |
| Patient 1 *p* | Cardiac arrest | CV (sudden cardiac death) | 93 | 128 |  |
| Patient 2 *p* | Tachycardia | CV (heart failure) | 310 | 318 |  |
| Deaths during safety follow-up (After November 4, 2016): Revusiran arm | | | | | |
| Patient 19* | Cardiac failure | CV (heart failure) | 351 | 404 |  |
| Patient 20 | Cardiac failure | CV (heart failure) | 316 | 419 |  |
| Patient 21 | Cardiac arrest | CV (sudden cardiac death) | 350 | 479 |  |
| Patient 22* | Cardiac failure congestive | CV (heart failure) | 366 | 422 |  |
| Patient 23 | Death | Undetermined | 415 | 553 |  |
| Deaths during safety follow-up (after November 4, 2016): Placebo arm | | | | |  |
| Patient 3 *p* | Low cardiac output syndrome | CV (heart failure) | 176 | 336 |  |
| Patient 4 *p* | Cardiac failure congestive | CV (heart failure) | 261 | 371 |  |
| Patient 5 *p* | Cardiac failure congestive | CV (heart failure) | 316 | 367 |  |
| Patient 6 *p* | Death | CV (sudden cardiac death) | 352 | 440 |  |
| Patient 7 *p* | Cardiac failure chronic | CV (heart failure) | 497 | 585 |  |

*This patient had an AE with a start date during the on-treatment period (prior to November 4, 2016) which led to death during the safety follow-up period (after November 4, 2016)
*AE* adverse event; *CV* cardiovascular, *p* placebo.

**Supplementary Table 2** Mean revusiran pharmacokinetic exposure parameter (C_max_) for all patients and by outcome status during treatment (pharmacokinetic population)

| Time, mean (SD) | C_max_ (µg/ml) All patients in the revusiran arm on-treatment | C_max_ (µg/ml) Patients in the revusiran arm who died on-treatment | C_max_ (µg/ml) Patients in the revusiran arm alive on-treatment |
| --- | --- | --- | --- |
| Baseline | *n* = 133 856 (546) | *n* = 17 1092 (749)^*^ | *n =*116 821 (505)^*^ |
| Month 6 | *n* = 72 1132 (1048) | *n* = 10 1478 (970)^†^ | *n* = 62 1076 (1057)^†^ |
| Month 12 | *n* = 17 1154 (588) | NA | NA |

Plasma revusiran C_max_ data were from the 2.5 ± 1 h post-dose time point
^*^*p =*0.20 (Kruskal–Wallis test between died versus alive)
^†^*p* = 0.13 (Kruskal–Wallis test between died versus alive)
*C_max_* maximum plasma concentration, *NA* not applicable, *SD* standard deviation

**Supplementary Table 3** Cardiac biomarkers and key echocardiogram parameters over time during the on-treatment period (modified intent-to-treat population)

|  | Placebo (*n =*66) | Revusiran (*n* = 140) |
| --- | --- | --- |
| Median (range) troponin I, µg/l *n* Baseline Month 6 | 40 0.14 (0.020–0.950) 0.15 (0.016–1.180) | 75 0.11 (0–1.660) 0.11 (0–3.300) |
| Median (range) NT-proBNP, pg/ml *n* Baseline Month 6 | 40 2698 (441–16,170) 3514 (595–28,450) | 70 2426 (84–12,280) 2812 (142–14,757) |
| Mean (SEM) intraventricular septum thickness, mm *n* Baseline Month 6 | 40 19 (0.4) 18 (0.4) | 75 18 (0.3) 18 (0.3) |
| Mean (SEM) left ventricular mass, g *n* Baseline Month 6 | 40 334.5 (11.6) 338.5 (10.5) | 74 335.3 (9.0) 323.2 (9.5) |
| Mean (SEM) left ventricular ejection fraction, % *n* Baseline  Month 6 | 38 50.8 (1.6) 49.7 (1.6) | 73 53.4 (1.4) 54.6 (1.3) |

*NT-proBNP* *N*-terminal prohormone of brain natriuretic peptide, *SEM* standard error of the mean

**Supplementary Fig. 1** Safety monitoring during safety follow-up period after study drug discontinuation. ^*^For patients with new onset or worsening peripheral neuropathy.
ECHO = echocardiogram; NYHA = New York Heart Association; PND score = polyneuropathy disability score


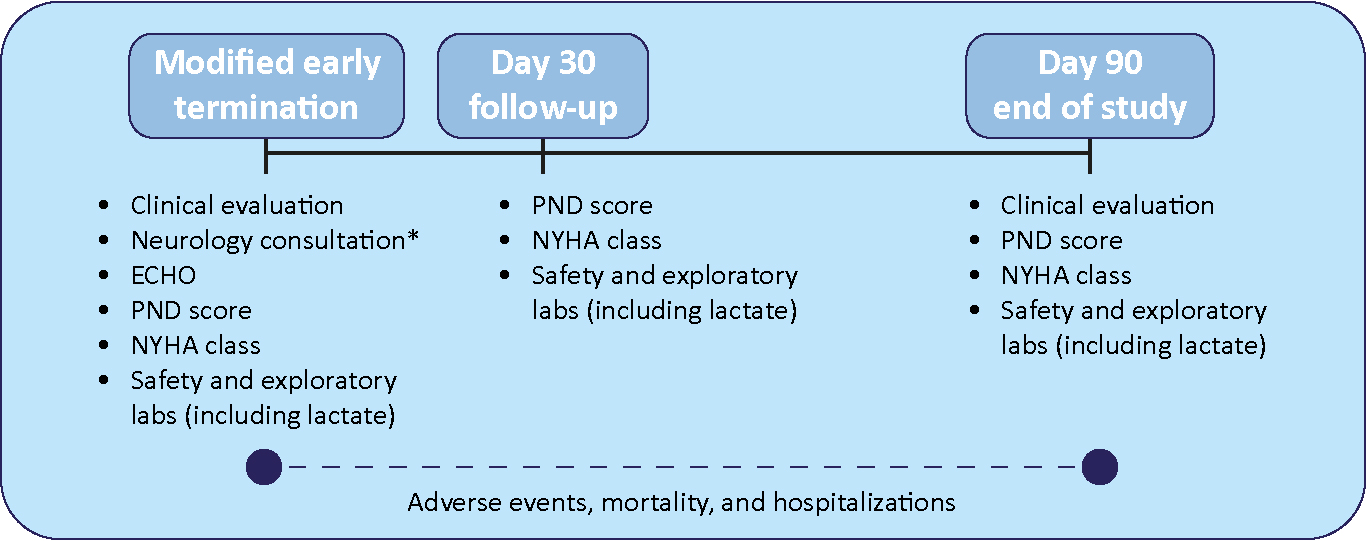


**Supplementary Fig. 2** Schematic of patient enrollment, randomization, and disposition. ^*^Completion of the modified early termination visit at the end of the safety follow-up period after treatment discontinuation. AE = adverse event; mITT = modified intent-to-treat


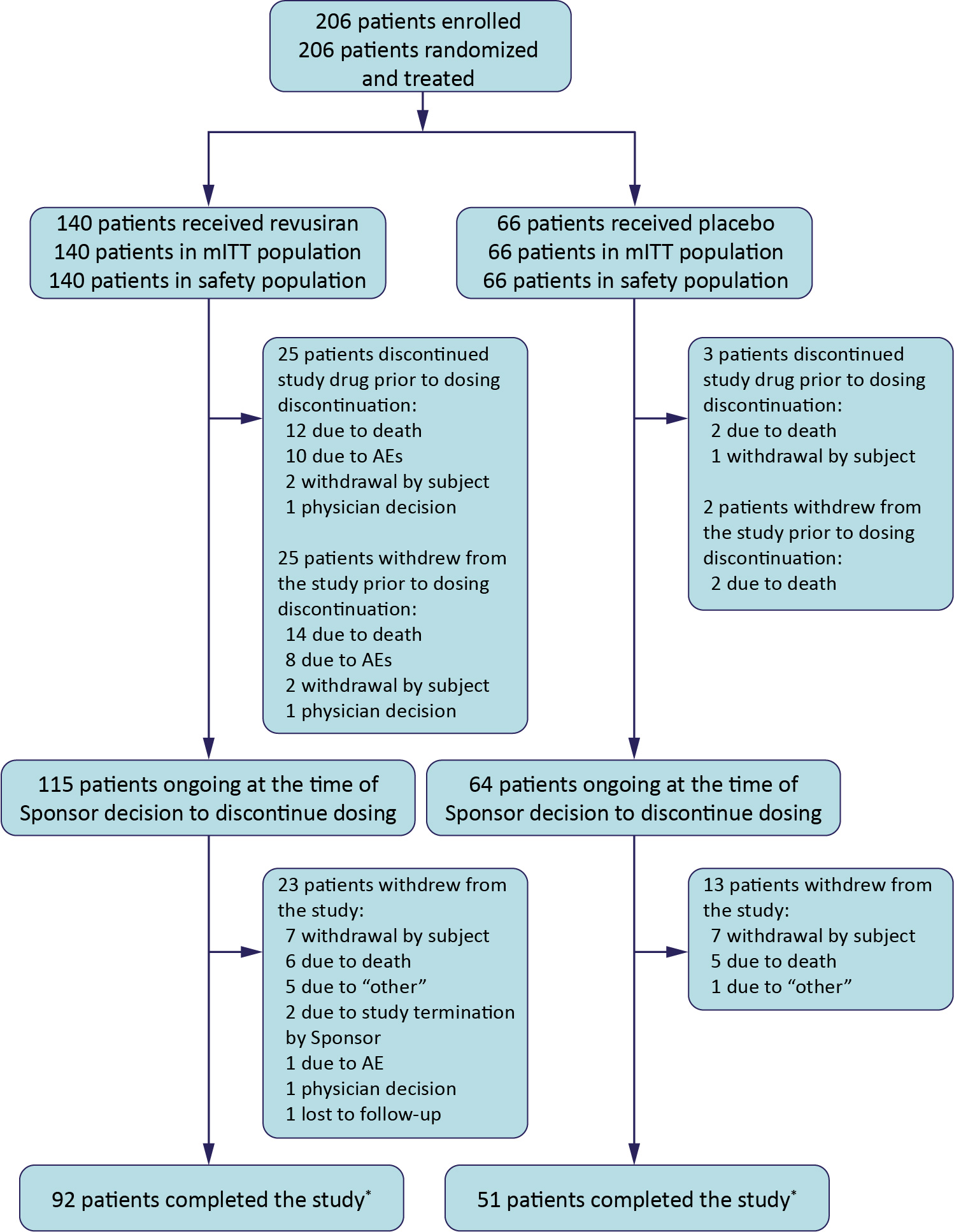


**Supplementary Fig. 3** All-cause mortality through end of safety follow-up period (modified intent-to-treat population). CI = confidence interval; HR = hazard ratio


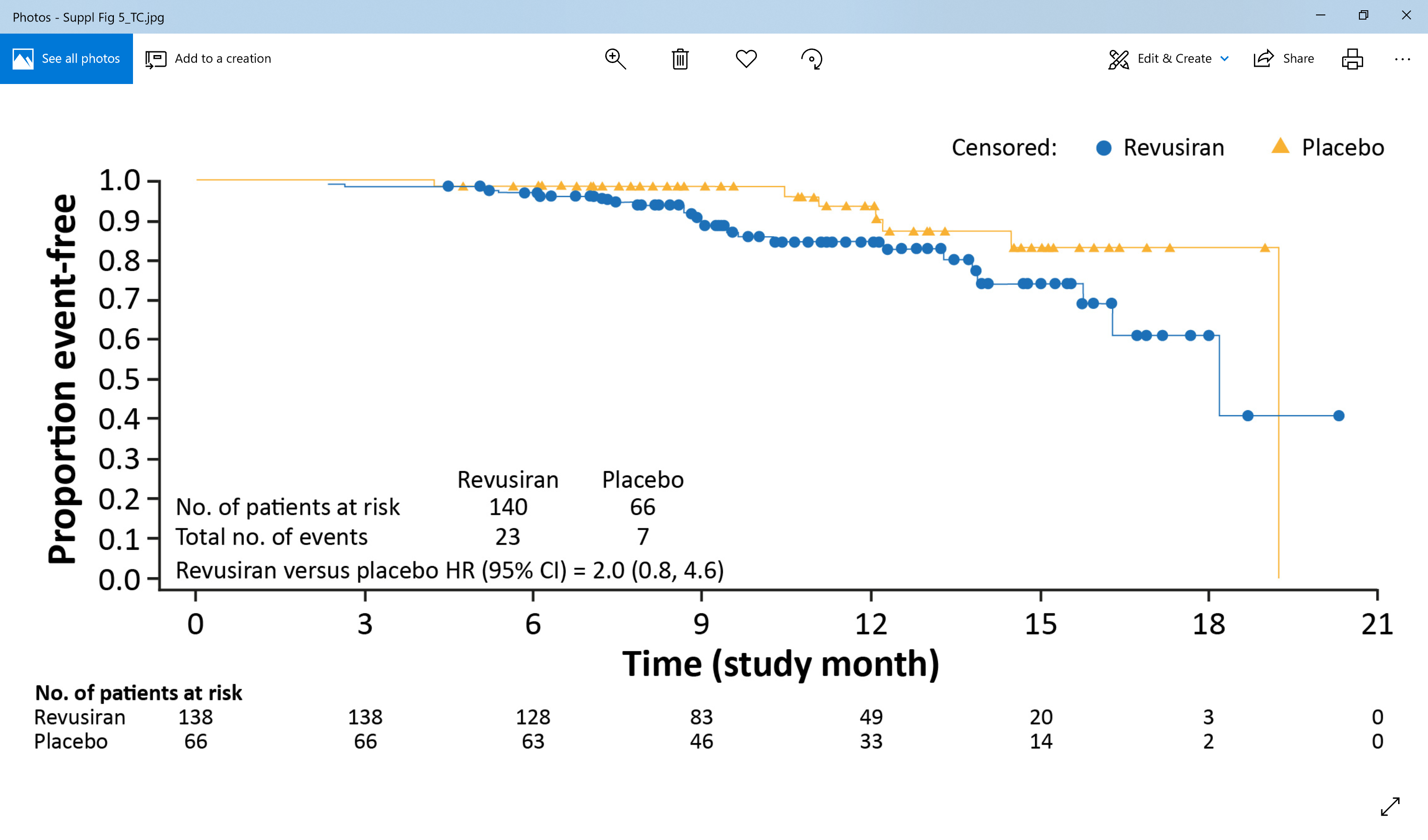


**Supplementary Fig. 4** Composite analysis of time to first heart failure hospitalization or cardiovascular mortality during the on-treatment period (modified intent-to-treat population). CI = confidence interval; HR = hazard ratio


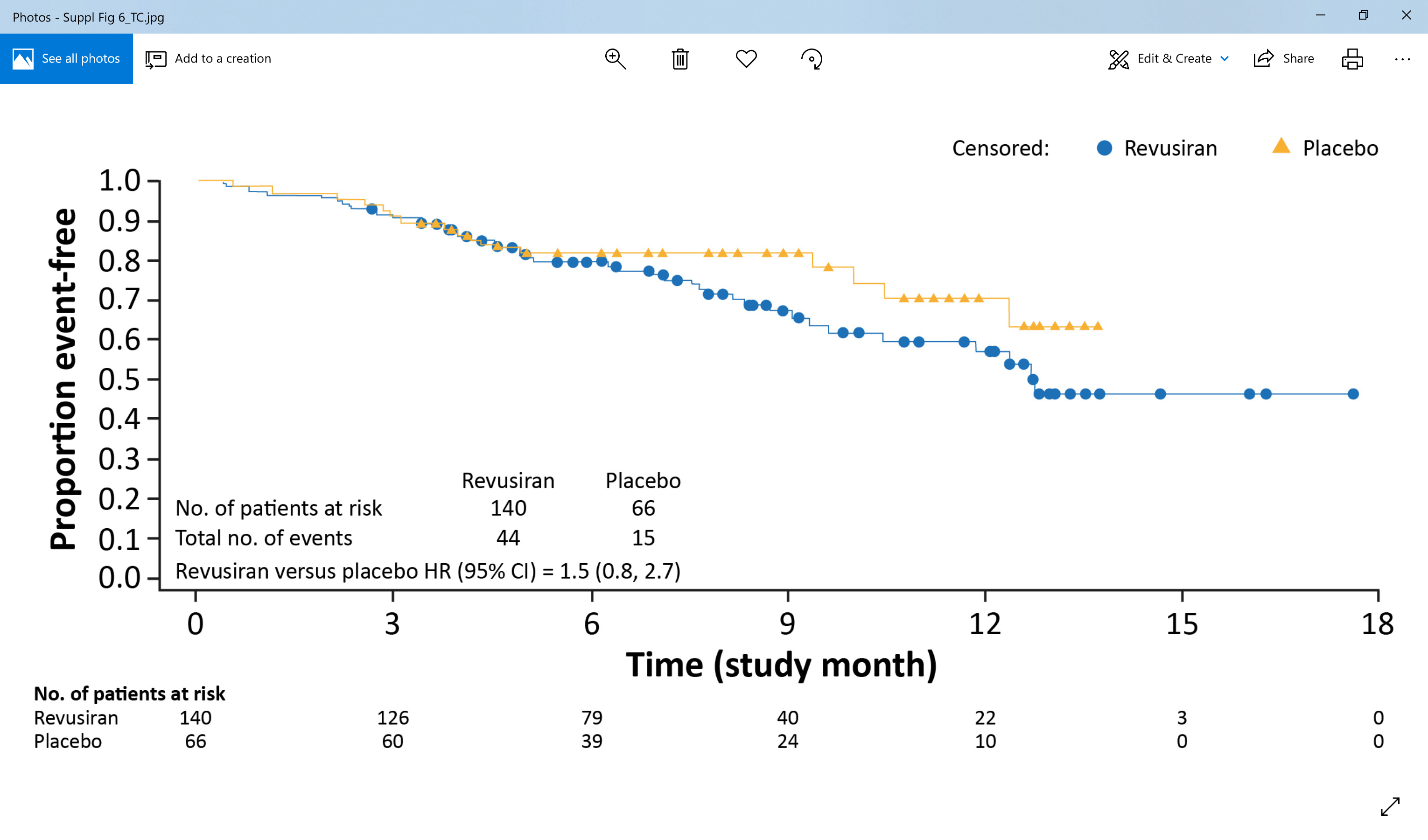
**Supplementary Fig. 5** Venous lactate levels relative to upper limit of normal at each scheduled visit by treatment arm during safety follow-up. ET = early termination; ULN = upper limit of normal


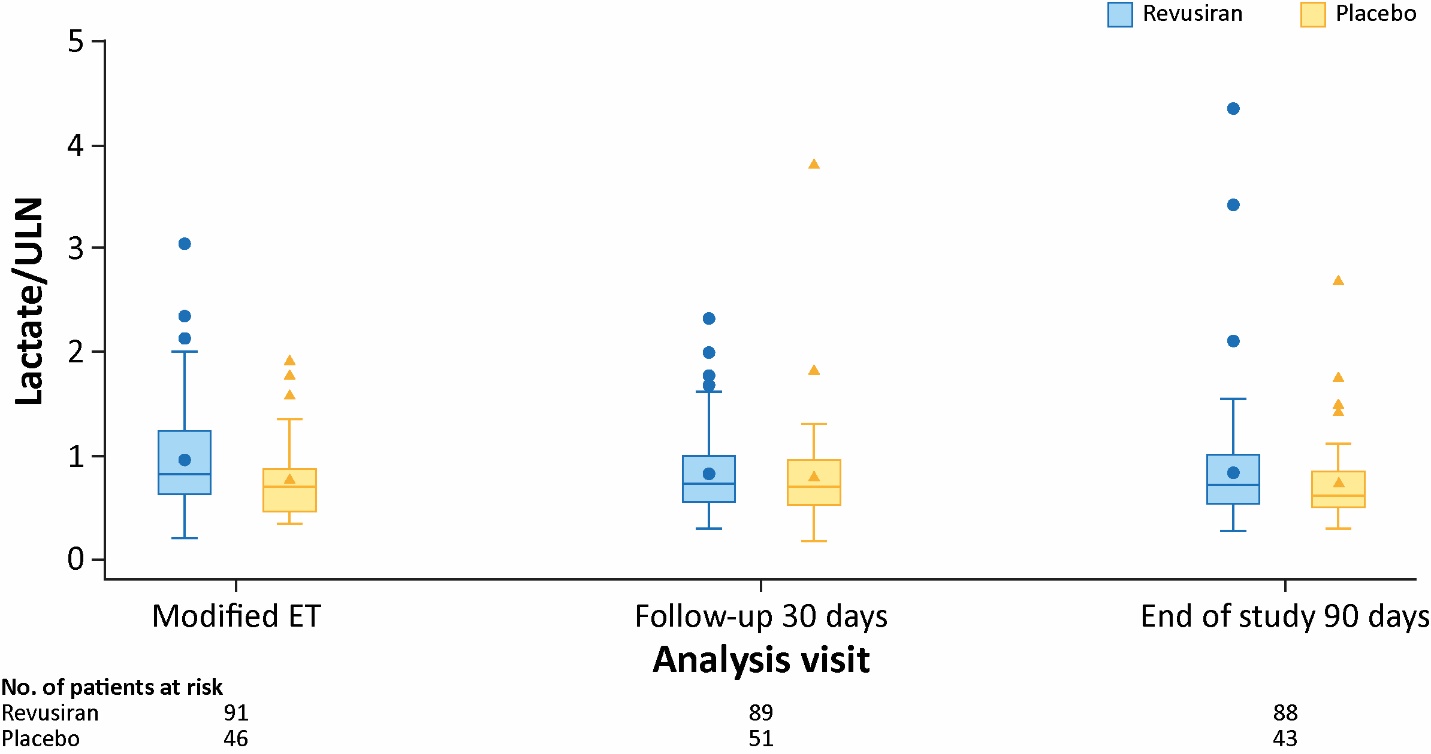


**Supplementary Fig. 6** Mean revusiran plasma concentration by eGFR category in the revusiran arm (safety population). Plasma concentration = plasma C_max_. Kruskal–Wallis test was performed to compare distributions of C_max_ between groups (eGFR: 30 to < 60, 60 to < 90, and ≥ 90 ml/min/1.73 m^2^) at each visit. C_max_ = maximum plasma concentration; eGFR = estimated glomerular filtration rate


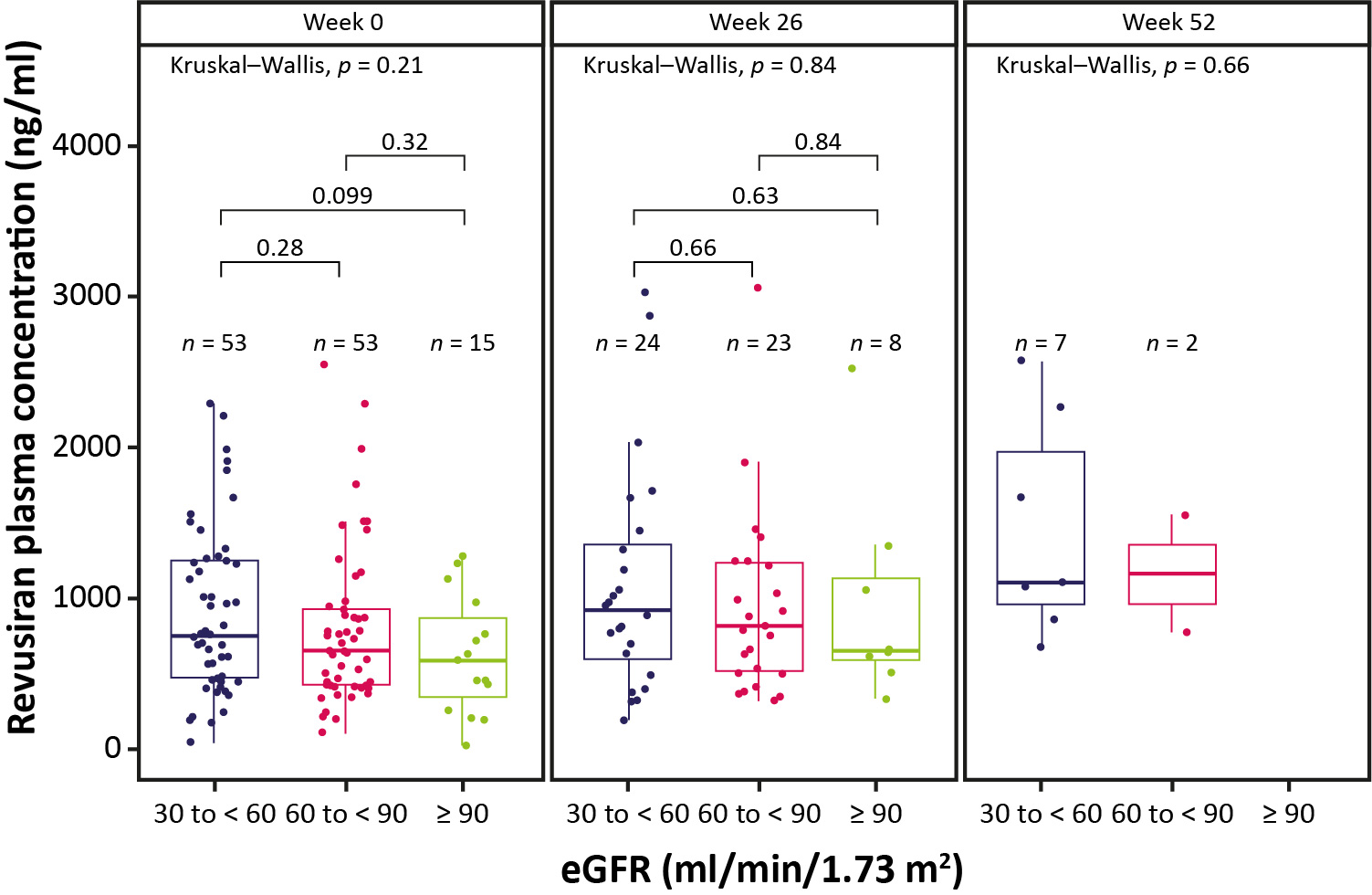


**Supplementary Fig. 7** TTR reduction by outcome during the on-treatment period (safety population). SEM = standard error of the mean; TTR = transthyretin


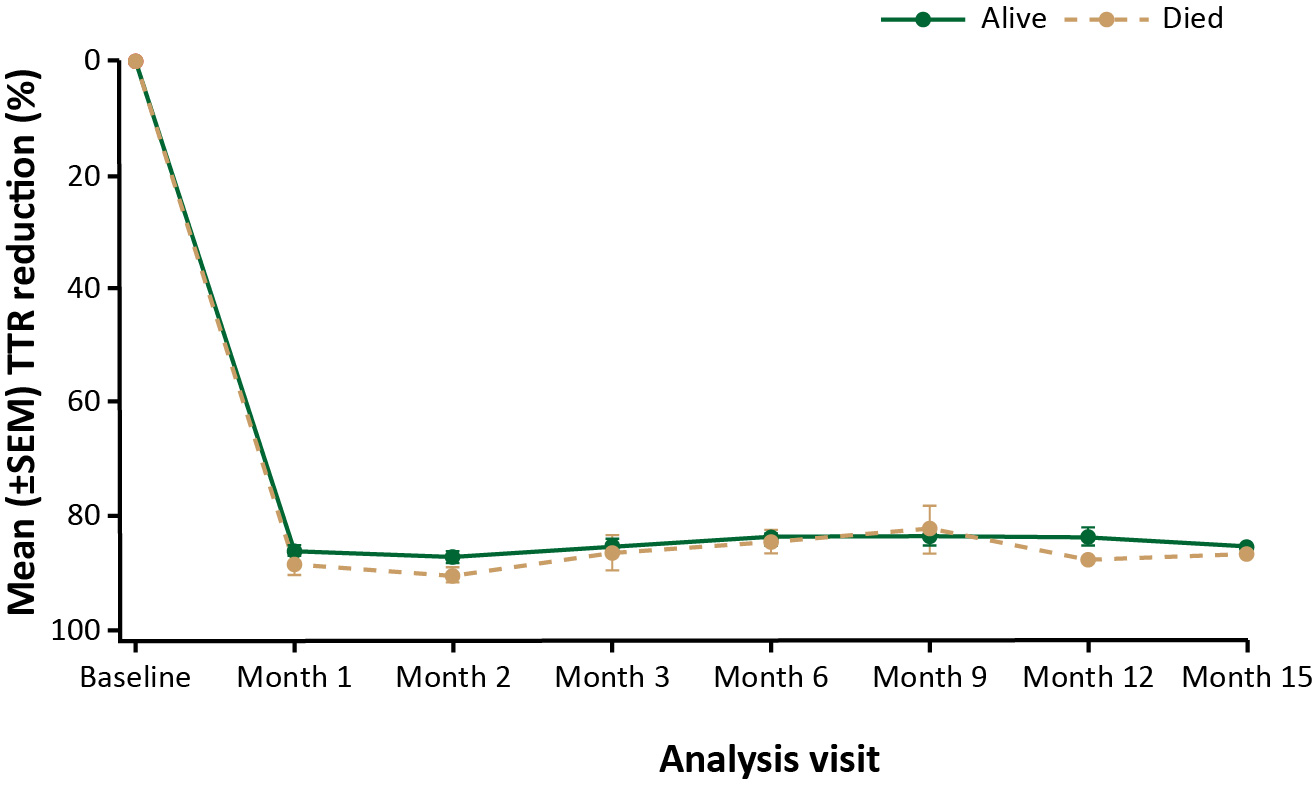

Supplement: Supplementary file 1 — (DOCX 2.58 mb) [file 10557_2019_6919_MOESM1_ESM.docx]
